# Identification of the synthetic cannabinoid‐type new psychoactive substance, CH‐PIACA, in seized material

**DOI:** 10.1002/dta.3333

**Published:** 2022-06-16

**Authors:** Daniel Pasin, Michael Nedahl, Christian Brinch Mollerup, Christian Tortzen, Lotte Ask Reitzel, Petur Weihe Dalsgaard

**Affiliations:** ^1^ Section of Forensic Chemistry, Department of Forensic Medicine, Faculty of Health and Medical Sciences University of Copenhagen Copenhagen Denmark; ^2^ Department of Chemistry University of Copenhagen Copenhagen Denmark

**Keywords:** drug seizure, NPS, synthetic cannabinoids

## Abstract

Synthetic cannabinoids (SCs) remain the largest class of new psychoactive substances (NPS), and while the number of NPS that are reported to the European Monitoring Centre for Drugs and Drug Addiction (EMCDDA) for the first time each year declines, the number of newly reported SCs still exceeds other NPS classes. This decline can be seen as a result of legislative changes by different jurisdictions which have sometimes transitioned to a more generalized approach when controlling substances by defining common structural scaffolds rather than explicit structures. While the consequences of such legislative changes have been expected over the years, the introduction of so‐called “class‐wide” bans puts further pressure on clandestine laboratories to synthesize compounds which are out of the scope of the legislation, and thus, these compounds are initially harder to detect and/or identify in the absence of analytical data. Recently, a SC with an indole‐3‐acetamide core‐linker scaffold, AD‐18 (i.e., ADB‐FUBIATA or ADB‐FUBIACA), was reported for the first time in China in 2021. Here, an additional cannabinoid with the indole‐3‐acetamide scaffold, *N*‐**c**yclo**h**exyl‐2‐(1‐**p**entyl‐1*H*‐**i**ndol‐3‐yl)**ac**et**a**mide (CH‐PIACA), is reported which was identified for the first time in a seized material in Denmark. Structural characterization was performed using gas chromatography–mass spectrometry (GC–MS), liquid chromatography‐high‐resolution mass spectrometry (LC‐HRMS), and nuclear magnetic resonance (NMR) spectroscopy.

## Graphical abstract

A new synthetic cannabinoid, *N*‐**c**yclo**h**exyl‐2‐(1‐**p**entyl‐1*H*‐**i**ndol‐3‐yl)**ac**et**a**mide (CH‐PIACA), with the indole‐3‐acetamide scaffold was identified in a seized material in Denmark and characterized using gas chromatography–mass spectrometry (GC–MS), ultrahigh performance liquid chromatography‐quadrupole time‐of‐flight‐mass spectrometry (UPLC‐QTOF‐MS), and nuclear magnetic resonance (NMR) spectroscopy. This marks the second synthetic cannabinoid with this scaffold that has been reported with further analogs anticipated to emerge in the near future.

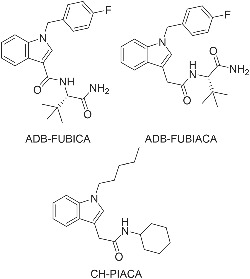

## 1 INTRODUCTION

**FIGURE 1:**
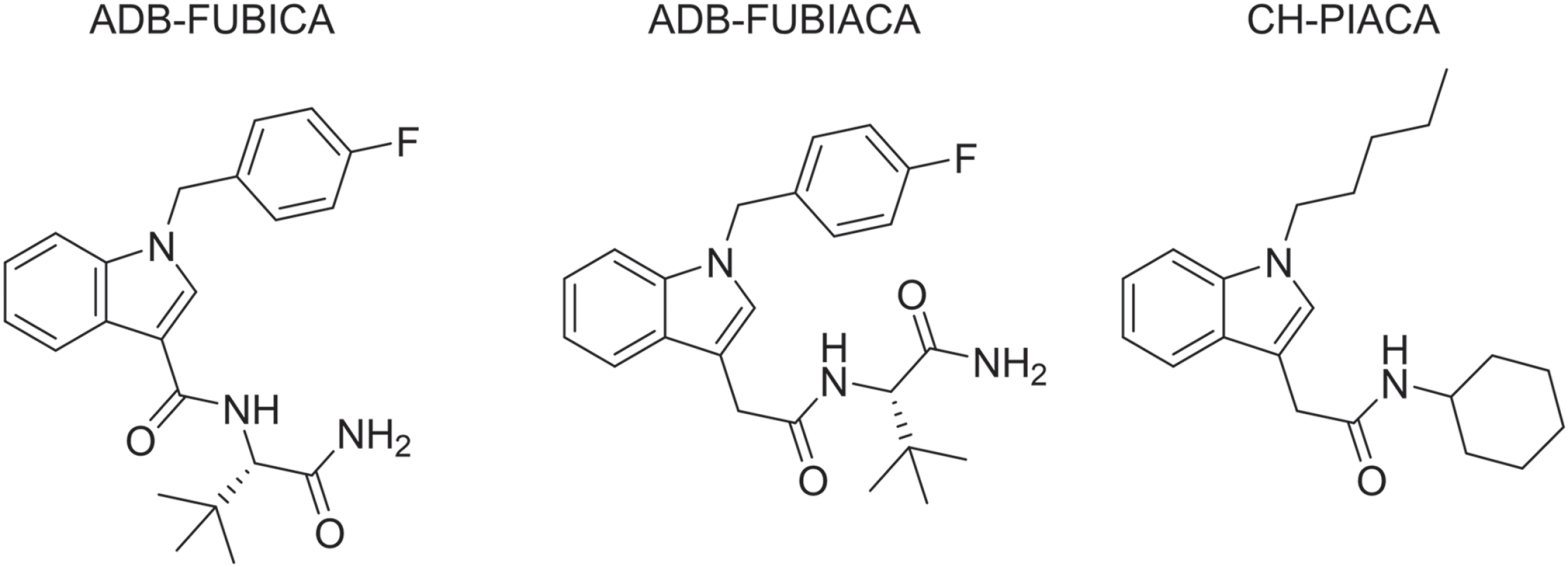
Structures of ADB‐FUBICA, ADB‐FUBIACA, and CH‐PIACA

The number of new psychoactive substances (NPS) that are reported for the first time to the European Monitoring Centre for Drugs and Drug Addiction (EMCDDA) has declined in recent years; however, synthetic cannabinoids (SCs) remain the largest NPS class, representing 209 of the 830 currently monitored NPS. Of the 46 NPS that were first reported in 2020, 11 were SCs followed closely by synthetic opioids (*n* = 10), a class which has seen a concerning growth of potent analogs in recent years.^1^ While this decline could be a result of many factors, it is believed that the changing legislative landscape is primarily responsible for this downturn. Traditionally, many countries enumerate individually controlled substances in the annexes of the legislation; however, some countries have now taken a more generalized approach and controlled substances based on common structural scaffolds.^2^, ^3^ For example, in 2016, Germany introduced the New Psychoactive Substance Act (NpSG) and its subsequent amendment in 2019 which controlled substances based on the well‐accepted premise that SCs can be considered a modular system containing a linked group (or head), linker, core and side chain (or tail).^4^ This legislation outlined common structural features within these groups that had been observed throughout the evolution of NPS, and thus, if a structure had a combination of these features, it would be ultimately controlled. This recently led to the emergence of unique tail substituents such as cyclobutylmethyl,^4^ cyclohexylsulfonyl, norbornyl methyl,^5^ and tosyl^6^ groups. More recently, China imposed a “class‐wide” ban on SCs from July 2021 based on seven of the main core groups found in existing SCs.^7^ Of these seven cores, most contain a ring system such as an indole, azaindole, indazole, pyrrole, and carbazole ring directly bonded to a carbonyl group or have the carbonyl group directly incorporated into the ring such as the γ‐carbolinone ring. This legislation differs from the NpSG as it only requires the core‐linker scaffold to be present for a substance to be controlled, therefore circumventing the issue of the emergence of novel tail or head groups. Consequently, this saw the re‐emergence of MDA‐19 (BZO‐HEXOXIZID) which had initially been identified in 2016^8^; however, now its pentyl (BZO‐POXIZID) and 5‐fluoropentyl (5F‐BZO‐POXIZID) analogs had also begun to emerge since they circumvent the class‐wide ban by having a distinct 2‐oxo‐indolin‐3‐ylidenehydrazine core‐linker scaffold.^7^ In addition, AD‐18 (i.e., ADB‐FUBIATA or ADB‐FUBIACA) was also identified. This compound only differs from ADB‐FUBICA, a controlled substance according to the Chinese class‐wide ban with an indole‐3‐carboxamide core‐linker scaffold, by the addition of a methylene linker between the indole ring and amido group (Figure 1). This minor yet effective workaround could result in the emergence of a series of analogs which contain the structural motifs observed in the last generations of SCs.

Here, the identification of a second SC with the indole‐3‐acetamide scaffold, *N*‐**c**yclo**h**exyl‐2‐(1‐**p**entyl‐1H‐**i**ndol‐3‐yl)**ac**et**a**mide and herein tentatively referred to as CH‐PIACA, is reported for the first time in a seized material confiscated by The Danish Customs Agency (Danish: Toldstyrelsen). Structural characterization was performed using gas chromatography–mass spectrometry (GC–MS), ultrahigh performance liquid chromatography‐quadrupole time‐of‐flight‐mass spectrometry (UPLC‐QTOF‐MS) and nuclear magnetic resonance (NMR) spectroscopy. Only recently has a certified reference material been made available for CH‐PIACA; therefore, this report serves to assist laboratories in detecting this substance by providing the necessary analytical data.

## 2 EXPERIMENTAL

### 2.1 Materials

LC–MS grade acetonitrile, methanol (MeOH) and water were purchased from Fisher Scientific (Leicestershire, UK). Formic acid, Leucine Enkephalin ≥95%, and deuterated dimethyl sulfoxide 99.5% were purchased from Sigma‐Aldrich (St. Louis, MO, USA). Ammonium formate ≥95% was purchased from VWR Chemicals (Radnor, PA, USA).

### 2.2 Sample preparation

The sample, consisting of 25.7 g of beige powder in a zip‐lock bag, was seized by The Danish Customs Agency at the International Post Office in Copenhagen Airport on February 1, 2022 and transported to the Section of Forensic Chemistry (Retskemisk Afdeling) on February 15 for analysis. Subsamples from the seized powder were made by combining three aliquots of approximately 1 g and homogenizing by mortar and pestle. Sample solutions for GC–MS and UPLC‐QTOF‐MS were prepared by dissolving 50 mg of the comminuted material in 5 ml MeOH followed by 10 min rotation and 5 min centrifugation. For GC–MS, the sample solution was diluted 1:10 using methanol, and mepivacaine was added as an internal standard with a final concentration of 26 mg/L. For UPLC‐QTOF‐MS, the sample solution was diluted 1:500 with 25% methanol + 1% formic acid and carbamazepine as an internal standard at a final concentration of 0.5 mg/L.

### 2.3 Instrumentation

#### 2.3.1 GC–MS

GC–MS analysis was carried out with an Agilent Technologies 7890A G3440A (Santa Clara, CA, USA) gas chromatograph coupled to an Agilent Technologies 5975C XL MS G3172A mass spectrometer. The instrument was operated in pulsed split mode (1:10), and the injection port temperature was maintained at 250°C. Separation of the 1 μl injection volume was performed using a HP‐5MS 19091S‐433 column (30 m × 0.25 mm i.d., 0.25 μm) with an initial temperature of 80°C then ramped 10°C/min to 310°C and held at 310°C for 15 min. The carrier gas (helium) was kept at constant flow of 0.6 ml/min. The transfer line, source, and quadrupole temperatures were 280°C, 230°C, and 150°C, respectively. The MS was operated in full scan mode in the range *m/z* 40–550. Acquired spectra were compared against an internal library, the European Network of Forensic Science Institutes Drugs Working Group (ENFSI DWG) shared database, and the NIST08 library.

#### 2.3.2 NMR spectroscopy

NMR spectroscopy was performed using a Bruker 500 MHz Avance III Fourier transform NMR spectrometer (Billerica, MA, USA) with a 5 mm observe cryoprobe, with 500.13 MHz for proton (^1^H) NMR, and 125.77 MHz for attached proton test carbon‐13 (APT^13^C) NMR. In addition, correlated spectroscopy (COSY), heteronuclear single quantum coherence (HSQC), and heteronuclear multiple bond correlation (HMBC) experiments were also performed. The chemical shifts were calibrated using the residual signal from the DMSO‐*d*_6_ as reference. The sample was prepared by dissolving 3 mg of the powder in DMSO‐*d*_6_ and filtering the clear solution into a NMR tube.

#### 2.3.3 UPLC‐QTOF‐MS

Separation was performed using a Waters Corporation ACQUITY I‐Class UPLC system (Manchester, UK) equipped with an ACQUITY UPLC HSS C18 column (150 mm × 2.1 mm, 1.8 μm) maintained at 50°C. The mobile phase was composed of solvent A (5 mM ammonium formate adjusted to pH 3.0) and solvent B (acetonitrile containing 0.1% formic acid) with a flow rate of 0.4 ml/min and injection volume of 1 μl. The initial mobile phase gradient was 13% B which was held for 0.5 min then ramped linearly to 50% B over 9.5 min followed by a second ramp from 50% to 95% B over 0.75 min and held for 1.5 min. The composition was returned to 13% B over 2.5 min for a total run time of 15 min. Mass spectrometry was performed using a Waters Corporation XEVO G2‐S QTOF‐MS (Manchester, UK). The mass spectrometer was operated in positive electrospray ionization (ESI+) mode. The nebulization gas was set to 800 L/h at a temperature of 400°C with the cone gas set to 20 L/h and source temperature of 150°C. Argon was used as the collision gas. Mass calibration was performed with sodium formate in propanol: water (9:1, v/v). Lock mass was used with leucine enkephalin as a reference mass at *m/z* 556.2766. Data were acquired using the data independent acquisition (DIA) technique, MS^E^, which had a low‐energy channel with a collision energy of 4 eV applied and high‐energy channel with a 10–40 eV ramp with a scan time of 0.2 s and a mass range from *m/z* 50 to 950.

## 3 RESULTS AND DISCUSSION

Routine GC–MS analysis yielded a major component at 22.156 min with a molecular ion at *m/z* 326, base peak fragment at *m/*z 200, and the two other main fragments, *m/z* 144 and *m/*z 130. There were also minor peaks observed at 7.255, 20.509, 21.726 and 23.998 min; however, these were considerably less abundant relative to the main component peak. Comparison of the electron ionization (EI) mass spectra against the selected libraries did not give any suitable matches. The minor components were not investigated further, with the TIC and EI mass spectra of each component provided in Figures S1–S5 of the supporting information (SI). Therefore, to elucidate the structure of the unknown major component, the sample was analyzed using NMR spectroscopy with ^1^H, ^13^C, COSY, HMBC, and HSQC experiments. Key NMR data from all experiments and their interpretation are listed in Table 1 with all spectra included in Figures S6–S10 in the SI. The triplet in the ^1^H spectrum at 0.84 ppm (position 12) was reminiscent of the methyl group of an alkyl chain. Couplings were tracked in COSY through positions 11, 10, 9, and 8, although the couplings to 10 overlapped with other signals in the aliphatic region. A triplet was observed at position 8 in the ^1^H spectrum, indicating a terminal alkyl chain. Through HSQC, a methyl and four methylene groups were found in the ^13^C spectrum, indicating a pentyl group. This was further supported by the HMBC spectrum where protons on position 10 correlated with carbons 8, 9, 11, and 12. In the aromatic region, protons on 4, 5, 6, and 7 coupled in the ^1^H and COSY spectra, while the proton on 2 was a singlet. This is consistent with a disubstituted indole. The quaternary carbons of the indole (i.e., positions 3a and 7a) were confirmed by HMBC correlations to the protons of the heterocyclic ring. The position of the pentyl group was also confirmed by HMBC as the protons on 8 correlated with carbons 2 and 7a.

**TABLE 1:** Interpretation of the NMR spectra

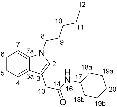						
Label no.	^13^C shift (ppm)	^1^H shift (ppm)	^1^H multiplicity, coupling constant (Hz)	Interpretation	COSY correlation	^1^H/^13^C‐HMBC correlation
2	126.9	7.18	s	Ar‐CH		3, 3a, 7a
3	108.6			Ar‐C		
3a	127.6			Ar‐C		
4	119.0	7.54	d, 7.8	Ar‐CH	5	3, 3a, 6, 7a
5	118.2	6.98	dt, 7.8; 1.0	Ar‐CH	4, 6	3a, 7
6	120.9	7.10	dt, 7,9; 1.0	Ar‐CH	5, 7	4, 7a
7	109.5	7.40	d, 7.9	Ar‐CH	6	3a, 5
7a	135.8			Ar‐C		
8	45.2	4.11	t, 7.0	C**H**_2_‐CH_2_	9	2, 7a, 9, 10
9	29.6	1.72	m	CH_2_	8, 10	8, 10, 11
10	28.5	1.20	m	CH_2_	9, 11	8, 9, 11, 12
11	21.8	1.29	m	CH_2_	10, 12	10, 12
12	13.9	0.84	t, 7.3	C**H**_3_‐CH_2_	11	10, 11
13	32.6	3.45	s	CH_2_		2, 3, 3a, 14
14	169.4			C=O		
16		7.80	Broad d	N**H**‐CH	17	
17	47.4	3.52	m	C‐H	16, 18a, 18b	
18a, 18b	32.5	1.14, 1.72	m,m	2x CH_2_	17, 19a, 19b	
19a, 19b	24.6	1.20, 1.66	m,m	2x CH_2_	18a, 18b, 20	
20	25.2	1.20, 1.54	m,m	CH_2_	19a, 19b	

*Note*: ^1^H multiplicity explanation: s: singlet, d: duplet, t: triplet. Multiplicities are described as observed.

Abbreviation: NMR, nuclear magnetic resonance.

Protons on 13 appeared to be a methylene group with no coupling to other protons. Through HMBC, 13 was found to be adjacent to the indole and carbon 14 which was in the amide ppm range. The HSQC spectrum revealed that three distinct carbons (18a, 18b, 19a, 19b, and 20) had nonequivalent protons attached, consistent with a saturated ring system. The proton on 17 gave rise to a multiplet, consistent with couplings to equatorial and axial protons on 18a, 18b, to which it coupled in COSY. The remaining protons overlapped each other and the pentyl chain in the aliphatic region. The number of protons and carbon found in the lower aliphatic region in the ^1^H and ^13^C spectra, respectively, support the presence of a cyclohexane ring. The cyclohexane proton on 17 showed a coupling in COSY to the proton on 16, which was believed to be a nitrogen due to signal broadening in the ^1^H spectrum and the lack of an HSQC correlation. This nitrogen was further believed to constitute the amide with carbon 14. The proton on 17 could theoretically correlate with carbon 14, but no correlation across the amide was observed, even when the HMBC experiment was rerun for an extended amount of time optimized for long range couplings.

From the NMR data, we thus identified fragments consisting of the following: 1‐pentyl‐3‐methylindole, a carbonyl group (part of an ester or amide based on the ^13^C ppm value), located 2 or 3 bonds away from protons on 13, and cyclohexyl amine where the amine is secondary. There is only one solution to assembling the molecule which is supported by the MS fragment data. Therefore, the structure was tentatively determined to be a substituted indole with a pentyl chain and *N*‐cyclohexylacetamide at the 1‐ and 3‐positions, respectively, namely, *N*‐cyclohexyl‐2‐(1‐pentyl‐1*H*‐indol‐3‐yl)acetamide (C_21_H_30_N_2_O; SMILES: C1(CCCCC1)NC(CC1=CN(C2=CC=CC=C12)CCCCC)=O; InChIKey = SYYOOLIGHZEOKJ‐UHFFFAOYSA‐N). The mass weight of the molecule (326.5 Da) was also consistent with mass of the component identified in GC–MS. All carbon attached protons were accounted for in HSQC, and the number of protons on carbons were confirmed by the ^13^C attached proton test.

**FIGURE 2:**
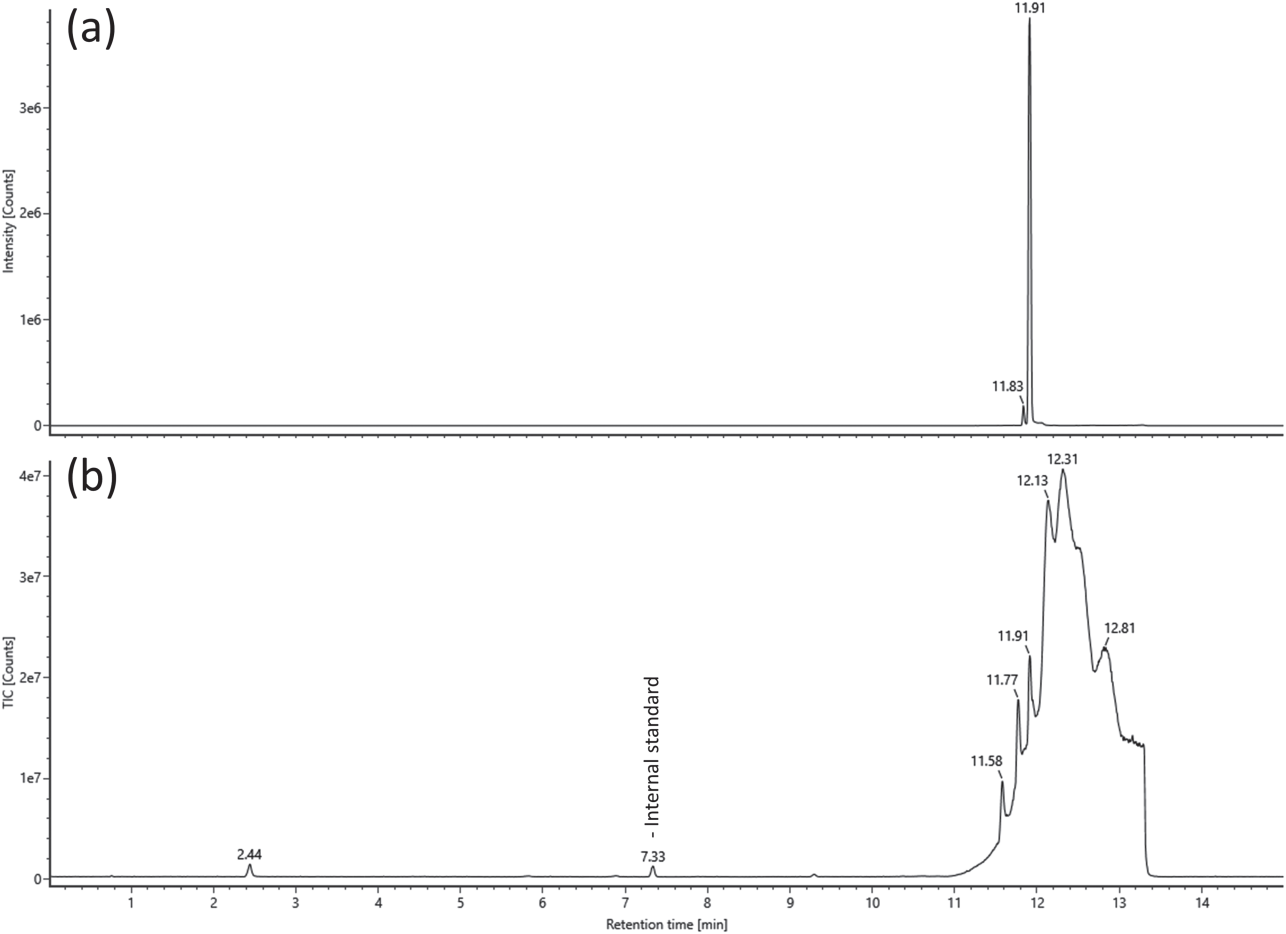
The extracted ion chromatogram for m/z 327.2431 corresponding to CH‐PIACA (a) and the total ion chromatogram of the seized material (b)

**FIGURE 3:**
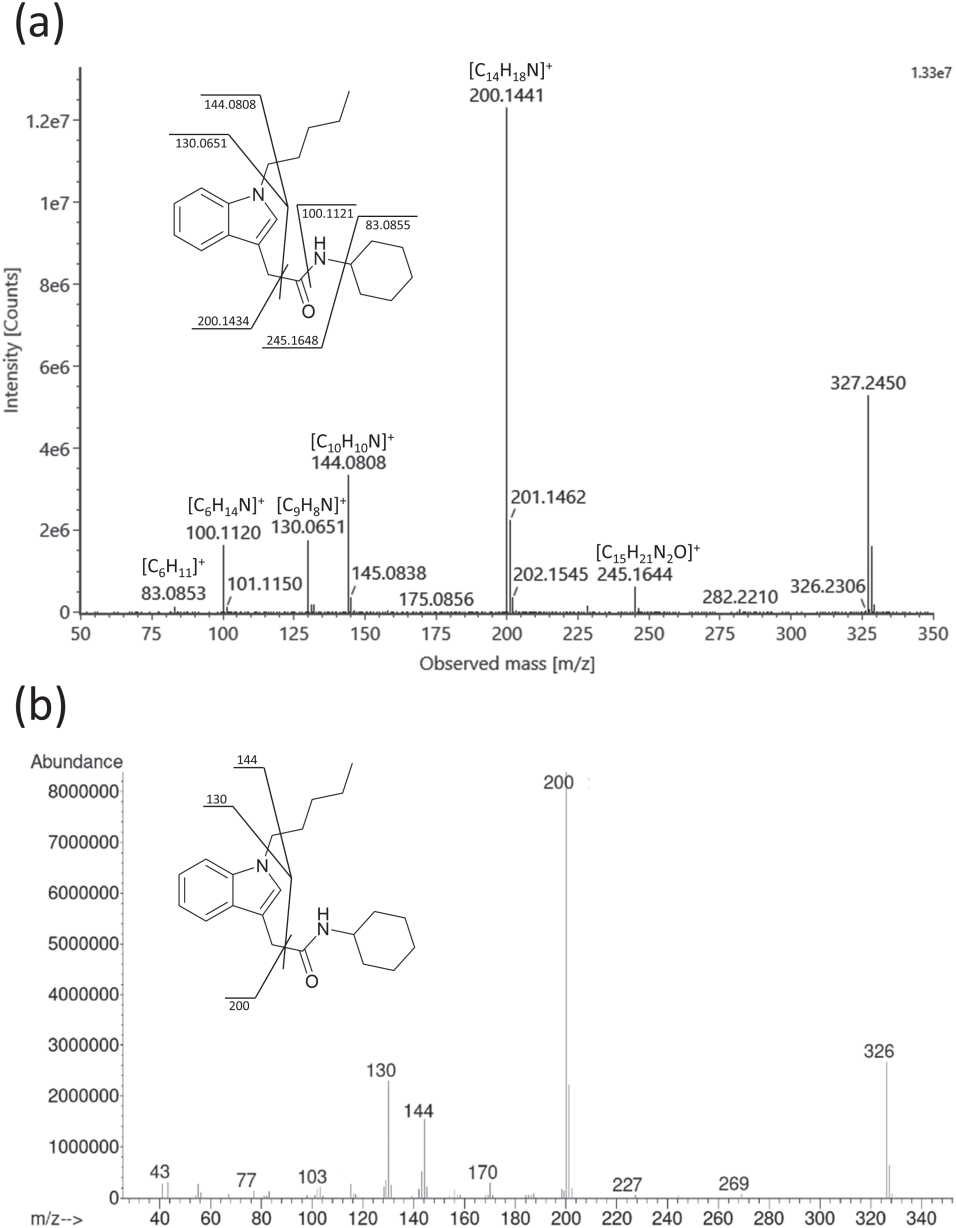
Electrospray ionization‐mass spectrometry (ESI‐MS/MS) spectrum of CH‐PIACA using a 10–40 eV collision energy ramp (a) and EI mass spectrum using a 70 eV collision energy (b) with the proposed fragment pathways annotated

The sample was also analyzed in parallel using UPLC‐QTOF‐MS which yielded components at 2.44, 5.83, 6.89, and 9.30; however, comparison of the MS/MS data (Figures S11–S15) for each component against an in‐house database containing 5476 compounds (including compounds from the NPS‐dedicated database, HighResNPS^9^) yielded no appreciable hits. In addition, the mass of the protonated molecule identified by NMR ([C_21_H_30_N_2_O + H]^+^; *m/z* 327.2431) was used to generate an extracted ion chromatogram which yielded a large component at 11.91 min (Figure 2a). This peak was not initially noticed as it eluted towards end of the run when the mobile phase composition was predominantly organic solvent and a large “wash‐out” peak is present (Figure 2b). Consequently, due to the use of DIA, the product ions in the high‐energy spectrum could not be confidently associated with the precursor ion due to many other co‐eluting substances. It should also be noted that SC‐type NPS tend to elute in this region of the chromatographic run. The sample was re‐analyzed; however, the precursor *m/z* was instead isolated prior to MS/MS experiments which yielded six product ions in the high‐energy MS/MS spectrum (Figure 3a) some of which were consistent with those observed in the EI mass spectrum (Figure 3b).

The product ions observed in the ESI‐MS/MS spectrum corresponded to the neutral loss of *N*‐cyclohexyl‐carboxamide (C_7_H_13_NO, 127.0997 Da) via cleavage of the bond between the α and β carbons to form *m/z* 200.1441 ([C_14_H_18_N]^+^, +3.5 ppm), while the minor product ion at *m/z* 245.1644 ([C_15_H_21_N_2_O]^+^, −2.0 ppm) corresponded to the loss of cyclohexene (C_6_H_10_, 82.0782 Da) from the amide nitrogen. The loss of the *N*‐cyclohexyl‐carboxamide was also followed by the loss of 1‐pentene (C_5_H_10_, 70.0783 Da) from the nitrogen of the indole ring to yield *m/z* 130.0651 ([C_9_H_8_N]^+^, 0.0 ppm), while *m/z* 100.1120 corresponded to the cyclohexylammonium cation ([C_6_H_14_N]^+^, −1.0 ppm). The product ion at *m/z* 144.0808 ([C_10_H_10_N]^+^, 0.0 ppm) was proposed to be due to the loss of both the *N*‐cyclohexyl‐carboxamide and cleavage of the pentyl chain to liberate 1‐butene (C_4_H_8_, 56.0626 Da). This fragment has also been observed in the mass spectra of previously studied *N*‐pentyl‐containing SCs using EI‐MS, which also suggest an α‐cleavage of the pentyl chain.^10^, ^11^, ^12^ However, this pathway has seldomly been reported for ESI‐MS/MS.^13^ The proposed structure was thus corroborated by both the ESI‐MS/MS and EI‐MS data.

A targeted search of the product ions was performed on HighResNPS, and while these product ions were present in the database, it is interesting to note that they were often independently associated with different classes. For example, *m/z* 144.0808 and 130.0651 are known common product ions in some substituted cathinones,^14^, ^15^ while *m/z* 100.1120 is associated with the formation of the 1,1‐diethylaziridinium cation in nitazene‐related compounds.^16^ Interestingly, *m/z* 200.1441 was reported as a minor product ion for JWH‐175, JWH‐201 and its positional isomer JWH‐250; however, these have seldom been reported in literature. JWH‐175 is a closely related analog to JWH‐018 with a methylene rather than a carbonyl linker between the indole and naphthalene rings. Therefore, the formation of m/z 200.1434 could be formed from the neutral loss of the naphthalene ring. Similarly, for JWH‐201 and JWH‐250, this could be formed by subsequent losses of the carbonyl oxygen and the 4‐ or 2‐methoxybenzyl head group, respectively. HighResNPS is currently a closed user group; however, access to the database can be granted by contacting the corresponding author.

The structure of SCs is often considered a combination of a tail, core, linker, and head group which in this case are represented by a pentyl chain, indole ring, acetamide linker, and cyclohexane ring, respectively. While the use of pentyl chains, indole rings, and cyclohexane rings have long been used in the evolution of SCs, this is the second report of a SC which contains an acetamide linker. Despite this, indole‐3‐acetamides have already been explored as potential cannabinoid receptor modulators with a number of 1‐ and 6‐substituted derivatives showing lower affinities for the cannabinoid receptor 1 (CB_1_) and 2 (CB_2_) compared with their indole‐3‐carboxamide analogs.^17^ More recently, Deventer et al.^18^ investigated the CB_1_ and CB_2_ activation potentials of the first indole‐3‐acetamide that were reported (i.e., ADB‐FUBIACA) and found that there was a considerable decrease in both potency and efficacy compared with its indole‐3‐carboxamide analog, AB‐FUBICA. In addition, cyclohexane rings have often been used as a tail group in the form of a cyclohexylmethyl (CHM) group bonded to the nitrogen atom in the 1‐position of the indole and indazole rings such as in AB‐CHMICA and AB‐CHMINACA, respectively. The use of cyclohexyl groups as a head group was investigated by Banister et al^19^ with the synthesis of SDB‐003 and its fluoro analog 5F‐SDB‐003; however, these have not been reported to the EMCDDA. Despite the use of a common tail group as a head group and the subtle modification of replacing the carboxamide linker with an acetamide linker, it yielded an ESI‐MS/MS spectrum that was less characteristic of other known SCs, which initially hindered its identification. This further highlights the need for data from GC–MS, UPLC‐QTOF‐MS, and NMR to be made available to laboratories to aid identification of emerging SCs.

## 4 CONCLUSION

A new SC with the indole‐3‐acetamide scaffold was identified in a seized material in Denmark and characterized using GC–MS, UPLC‐QTOF‐MS and NMR spectroscopy. This marks the second SC with this scaffold that has been reported with further analogs anticipated to emerge in the near future.

## Supporting information


**Figure S1.** GC–MS TIC of the seized material
**Figure S2.** EI‐MS of the unknown peak at 7.255 min
**Figure S3.** EI‐MS of the unknown peak at 20.509 min
**Figure S4.** EI‐MS of the unknown peak at 21.726 min
**Figure S5.** EI‐MS of the unknown peak at 23.998 min
**Figure S6.** ^1^H spectrum of CH‐PIACA in DMSO‐*d*_6_
**Figure S7.** COSY spectrum of CH‐PIACA in DMSO‐*d*_6_
**Figure S8.** APT^13^C spectrum of CH‐PIACA in DMSO‐*d*_6_
**Figure S9.** HSQC spectrum of CH‐PIACA in DMSO‐*d*_6_
**Figure S10.** HMBC spectrum of CH‐PIACA in DMSO‐*d*_6_
**Figure S11.** UPLC‐QTOF‐MS TIC of the seized material
**Figure S12.** ESI‐MS of the unknown peak at 2.44 min
**Figure S13.** ESI‐MS of the unknown peak at 5.82 min
**Figure S14.** ESI‐MS of the unknown peak at 6.88 min
**Figure S15.** ESI‐MS of the unknown peak at 9.29 min

## Data Availability

The data that support the findings of this study are available in the supporting information of this article.

## References

[1] European Monitoring Centre for Drugs and Drug Addiction . European Drug Report 2021: Trends and Developments. https://www.emcdda.europa.eu/publications/edr/trends-developments/2021_en. Accessed March 25, 2022.

[2] van Amsterdam J , Nutt D , van den Brink W . Generic legislation of new psychoactive drugs. J Psychopharmacol. 2013;27(3):317‐324. doi:10.1177/0269881112474525 23343598

[3] Peacock A , Bruno R , Gisev N , et al. New psychoactive substances: challenges for drug surveillance, control, and public health responses. Lancet. 2019;394(10209):1668‐1684. doi:10.1016/S0140-6736(19)32231-7 31668410

[4] Halter S , Pulver B , Wilde M , et al. Cumyl‐CBMICA: A new synthetic cannabinoid receptor agonist containing a cyclobutyl methyl side chain. Drug Test Anal. 2021;13(1):208‐216. doi:10.1002/dta.2942 33037749

[5] Pulver B , Riedel J , Schönberger T , et al. Comprehensive structural characterisation of the newly emerged synthetic cannabimimetics Cumyl‐BC[2.2.1]HpMeGaClone, Cumyl‐BC[2.2.1]HpMINACA, and Cumyl‐BC[2.2.1]HpMICA featuring a norbornyl methyl side chain. Forensic Chem. 2021;26:100371. doi:10.1016/j.forc.2021.100371

[6] Pulver B , Schönberger T , Weigel D , et al. Structure elucidation of the novel synthetic cannabinoid Cumyl‐Tosyl‐Indazole‐3‐Carboxamide (Cumyl‐TsINACA) found in illicit products in Germany. Drug Test Anal. 2022;1‐20. doi:10.1002/dta.3261 35338591

[7] European Monitoring Centre for Drugs and Drug Addiction . EMCDDA – Europol 2016 Annual Report on the implementation of Council Decision 2005/387/JHA. https://www.emcdda.europa.eu/system/files/publications/4724/TDAN17001ENN_PDFWEB.pdf. Accessed May 5, 2022.

[8] Liu C‐M , Hua Z‐D , Jia W , Li T . Identification of AD‐18, 5F‐MDA‐19, and pentyl MDA‐19 in seized materials after the class‐wide ban of synthetic cannabinoids in China. Drug Test Anal. 2022;14(2):307‐316. doi:10.1002/dta.3185 34694738

[9] Mardal M , Andreasen MF , Mollerup CB , et al. HighResNPS.com: an online crowd‐sourced HR‐MS database for suspect and non‐targeted screening of new psychoactive substances. J Anal Toxicol. 2019;43(7):520‐527. doi:10.1093/jat/bkz030 31095696

[10] Shevyrin V , Melkozerov V , Nevero A , et al. Identification and analytical characteristics of synthetic cannabinoids with an indazole‐3‐carboxamide structure bearing a N‐1‐methoxycarbonylalkyl group. Anal Bioanal Chem. 2015;407(21):6301‐6315. doi:10.1007/s00216-015-8612-7 25893797

[11] Thaxton A , Belal TS , Smith F , DeRuiter J , Abdel‐Hay KM , Clark CR . Mass spectral studies on 1‐n‐pentyl‐3‐(1‐naphthoyl)indole (JWH‐ 018), three deuterium‐labeled analogues and the inverse isomer 1‐naphthoyl‐3‐n‐pentylindole. Rapid Commun Mass Spectrom. 2015;29(9):871‐877. doi:10.1002/rcm.7171 26377015

[12] Xing Y , Xu X , Liu X , Xu B , Ma Q , Lei H . Study on the mass fragmentation pathway of the synthetic cannabinoids JWH‐018 and JWH‐073. Int J Mass Spectrom. 2015;379:139‐145. doi:10.1016/j.ijms.2015.01.007

[13] Staeheli SN , Poetzsch M , Veloso VP , et al. In vitro metabolism of the synthetic cannabinoids CUMYL‐PINACA, 5F–CUMYL‐PINACA, CUMYL‐4CN‐BINACA, 5F–CUMYL‐P7AICA and CUMYL‐4CN‐B7AICA. Drug Test Anal. 2018;10(1):148‐157. doi:10.1002/dta.2298 28885775

[14] Fornal E . Formation of odd‐electron product ions in collision‐induced fragmentation of electrospray‐generated protonated cathinone derivatives: aryl α‐primary amino ketones. Rapid Commun Mass Spectrom. 2013;27(16):1858‐1866. doi:10.1002/rcm.6635 23857931

[15] Reitzel LA , Dalsgaard PW , Müller IB , Cornett C . Identification of ten new designer drugs by GC‐MS, UPLC‐QTOF‐MS, and NMR as part of a police investigation of a Danish Internet company. Drug Test Anal. 2012;4(5):342‐354. doi:10.1002/dta.358 22102551

[16] Vandeputte MM , Van Uytfanghe K , Layle NK , St. Germaine DM , Lula DM , Stove CP . Synthesis, chemical characterization, and μ‐opioid receptor activity assessment of the emerging group of “nitazene” 2‐benzylbenzimidazole synthetic opioids. ACS Chem Nerosci. 2021;12(7):1241‐1251. doi:10.1021/acschemneuro.1c00064 33759494

[17] Pasquini S , Mugnaini C , Ligresti A , et al. Design, synthesis, and pharmacological characterization of indol‐3‐ylacetamides, indol‐3‐yloxoacetamides, and indol‐3‐ylcarboxamides: potent and selective CB2 cannabinoid receptor inverse agonists. J Med Chem. 2012;55(11):5391‐5402. doi:10.1021/jm3003334 22548457

[18] Deventer MH , Van Uytfanghe K , Vinckier IMJ , Reniero F , Guillou C , Stove CP . A new cannabinoid receptor 1 selective agonist evading the 2021 “China ban”: ADB‐FUBIATA. Drug Test Anal. 2022; 1‐6. doi:10.1002/dta.3285 35570246

[19] Banister SD , Stuart J , Kevin RC , et al. Effects of bioisosteric fluorine in synthetic cannabinoid designer drugs JWH‐018, AM‐2201, UR‐144, XLR‐11, PB‐22, 5F‐PB‐22, APICA, and STS‐135. ACS Chem Nerosci. 2015;6(8):1445‐1458. doi:10.1021/acschemneuro.5b00107 25921407

