# Supplementary material for: Identification of the synthetic cannabinoid‐type new psychoactive substance, CH‐PIACA, in seized material
Source: Drug Test Anal. 2022 Jun 16;14(9):1645–51. doi: 10.1002/dta.3333 (PMC9544820; doi:10.1002/dta.3333)
Supplement: Supplementary file 1 — Figure S1. GC–MS TIC of the seized material Figure S2. EI‐MS of the unknown peak at 7.255 min Figure S3. EI‐MS of the unknown peak at 20.509 min Figure S4. EI‐MS of the unknown peak at 21.726 min Figure S5. EI‐MS of the unknown peak at 23.998 min Figure S6. 1H spectrum of CH‐PIACA in DMSO‐d 6 Figure S7. COSY spectrum of CH‐PIACA in DMSO‐d 6 Figure S8. APT13C spectrum of CH‐PIACA in DMSO‐d 6 Figure S9. HSQC spectrum of CH‐PIACA in DMSO‐d 6 Figure S10. HMBC spectrum of CH‐PIACA in DMSO‐d 6 Figure S11. UPLC‐QTOF‐MS TIC of the seized material Figure S12. ESI‐MS of the unknown peak at 2.44 min Figure S13. ESI‐MS of the unknown peak at 5.82 min Figure S14. ESI‐MS of the unknown peak at 6.88 min Figure S15. ESI‐MS of the unknown peak at 9.29 min [file DTA-14-1645-s001.docx]

**Supporting Information For**

**Identification of the synthetic cannabinoid-type new psychoactive substance, CH-PIACA, in seized material**

Daniel Pasin^1^, Michael Nedahl^1,^*, Christian Brinch Mollerup^1^, Christian Tortzen^2^, Lotte Ask Reitzel^1^, Petur Weihe Dalsgaard^1^

^1^Section of Forensic Chemistry, Department of Forensic Medicine, University of Copenhagen, Frederik V’s Vej 11, DK-2100 Copenhagen, Denmark

^2^Department of Chemistry, University of Copenhagen, Universitetsparken 5, DK-2100 Copenhagen, Denmark

*Corresponding Author:

Michael Nedahl

Section of Forensic Chemistry 
Department of Forensic Medicine 
University of Copenhagen 
Copenhagen, Denmark 
Email: Michael.nedahl@sund.ku.dk

**Figures**

**Figure S1. GC-MS TIC of the seized material** 3 **Figure S2. EI-MS of the unknown peak at 7.255 min** 4
**Figure S3.** **EI-MS of the unknown peak at 20.509 min** 5
**Figure S4**. **EI-MS of the unknown peak at 21.726 min** 6
**Figure S5. EI-MS of the unknown peak at 23.998 min** 7

**Figure S6. ^1^H spectrum of CH-PIACA in DMSO-*d*_6_** 8

**Figure S7. COSY spectrum of CH-PIACA in DMSO-*d*_6_** 9

**Figure S8. APT^13^C spectrum of CH-PIACA in DMSO-*d*_6_** 10

**Figure S9. HSQC spectrum of CH-PIACA in DMSO-*d*_6_** 11

**Figure S10. HMBC spectrum of CH-PIACA in DMSO-*d*_6_** 12

**Figure S11. UPLC-QTOF-MS TIC of the seized material** 13

**Figure S12. ESI-MS of the unknown peak at 2.44 min** 14

**Figure S13. ESI-MS of the unknown peak at 5.82 min** 15

**Figure S14. ESI-MS of the unknown peak at 6.88 min** 16

**Figure S15. ESI-MS of the unknown peak at 9.29 min** 17


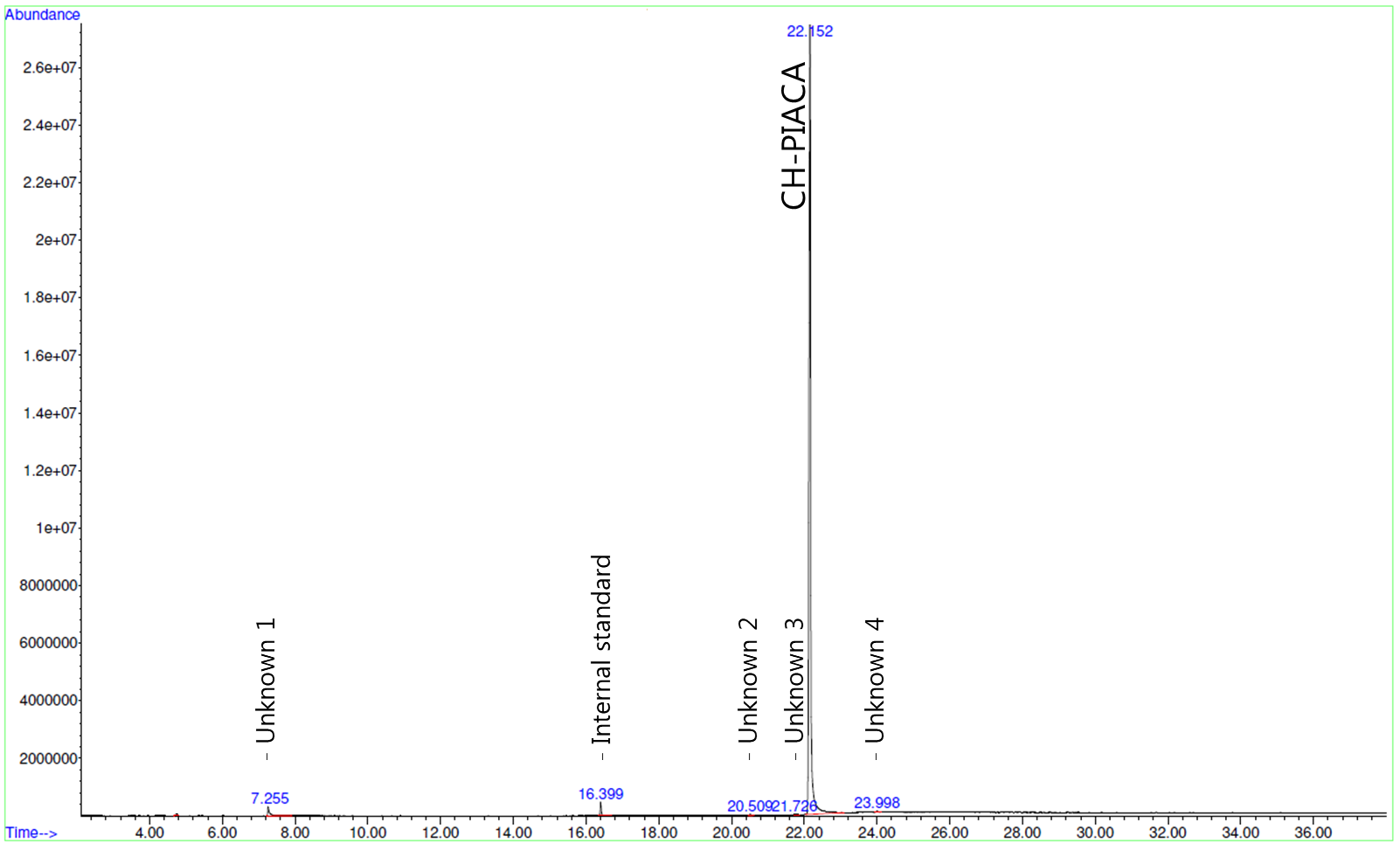


**Figure S1**. GC-MS TIC of the seized material.


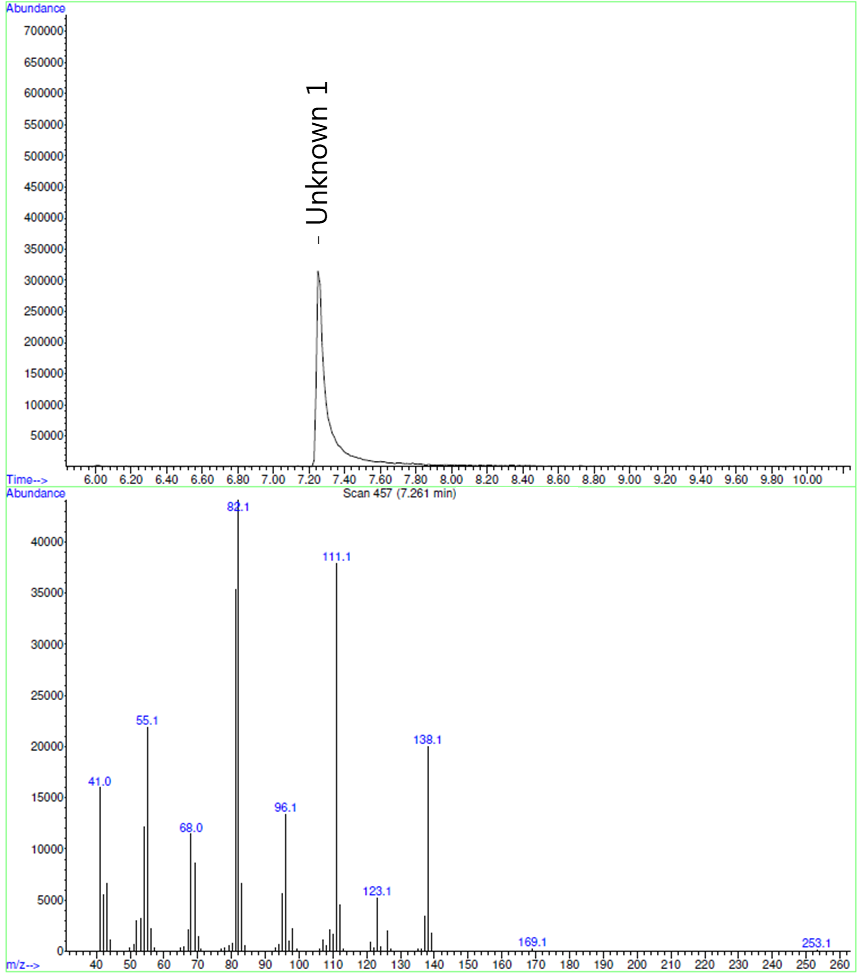


**Figure S2**. EI-MS of the unknown peak at 7.255 min


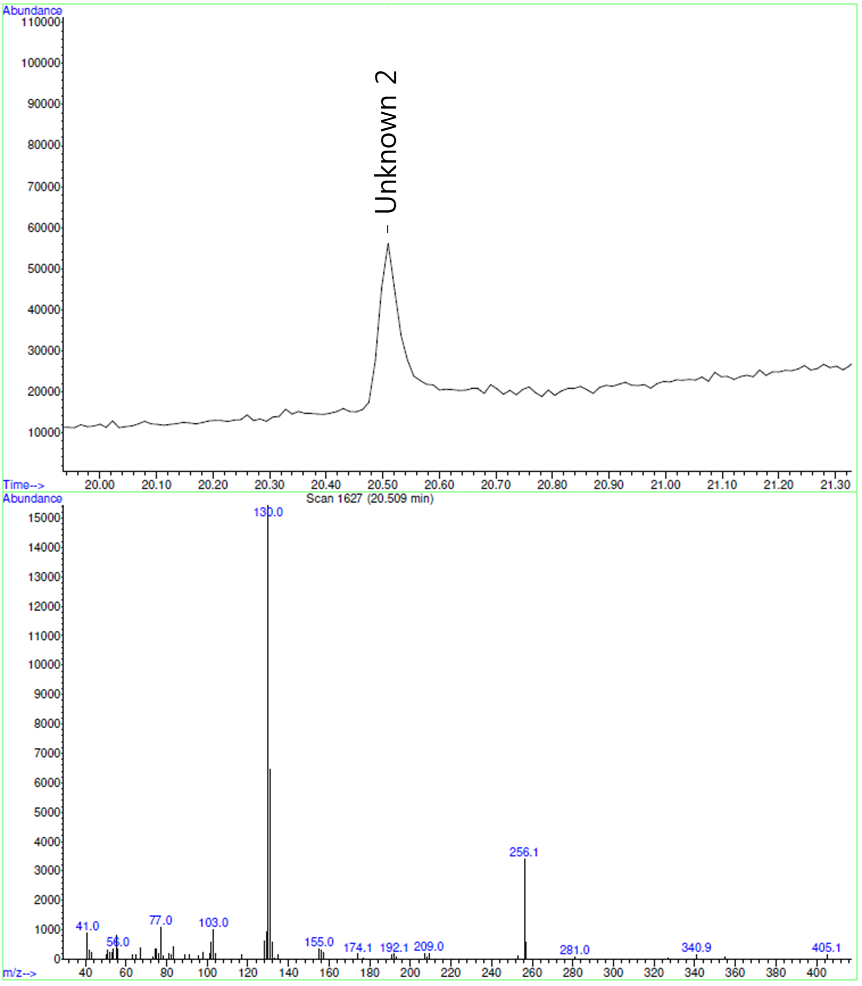


**Figure S3.** EI-MS of the unknown peak at 20.509 min

**
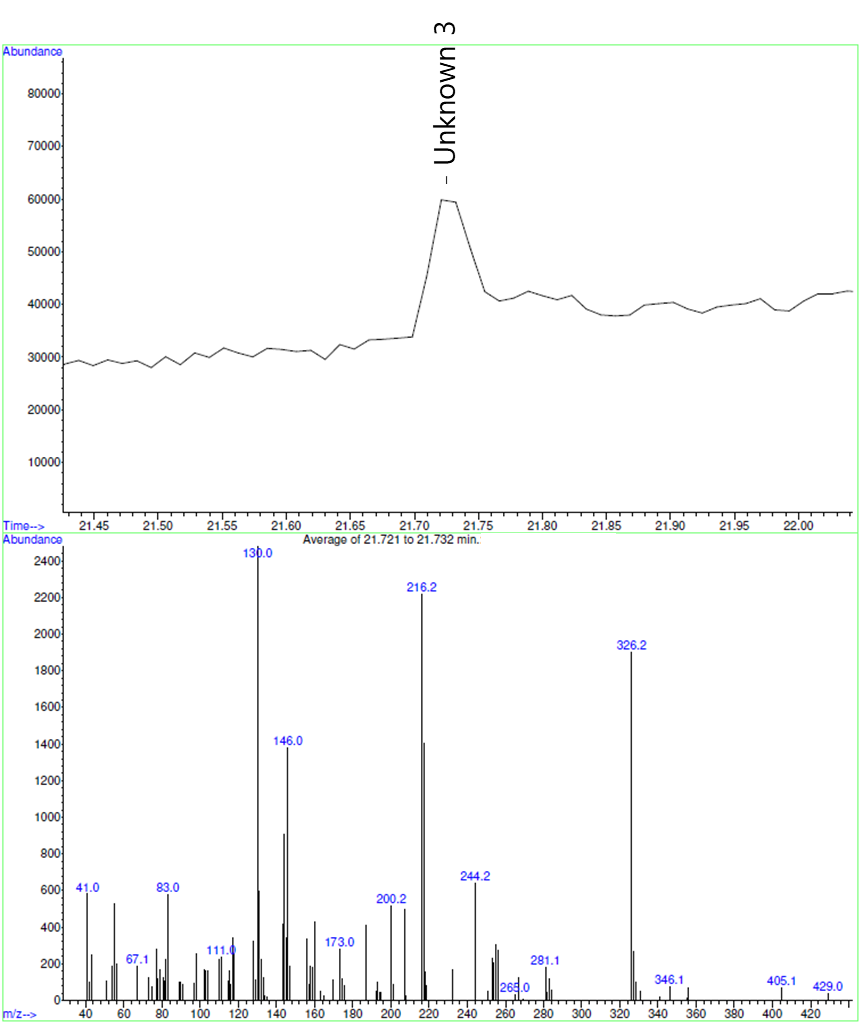
**

**Figure S4.** EI-MS of the unknown peak at 21.726 min**.**

**
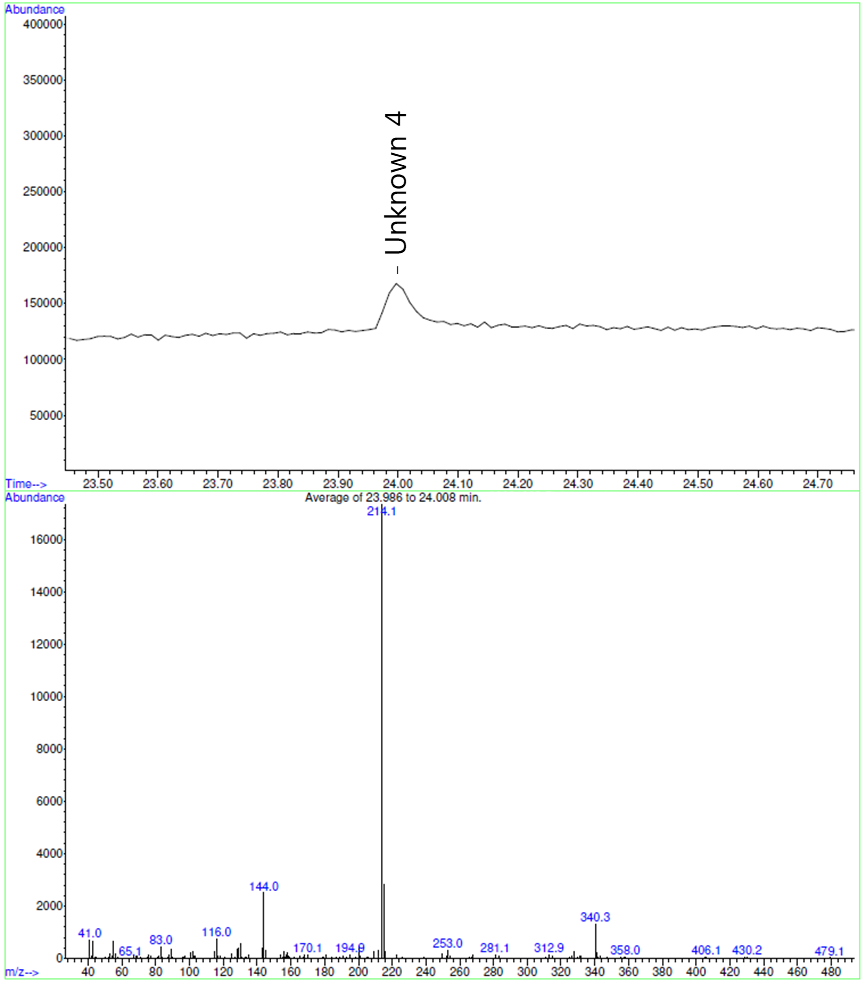
**

**Figure S5.** EI-MS of the unknown peak at 23.998 min**.**


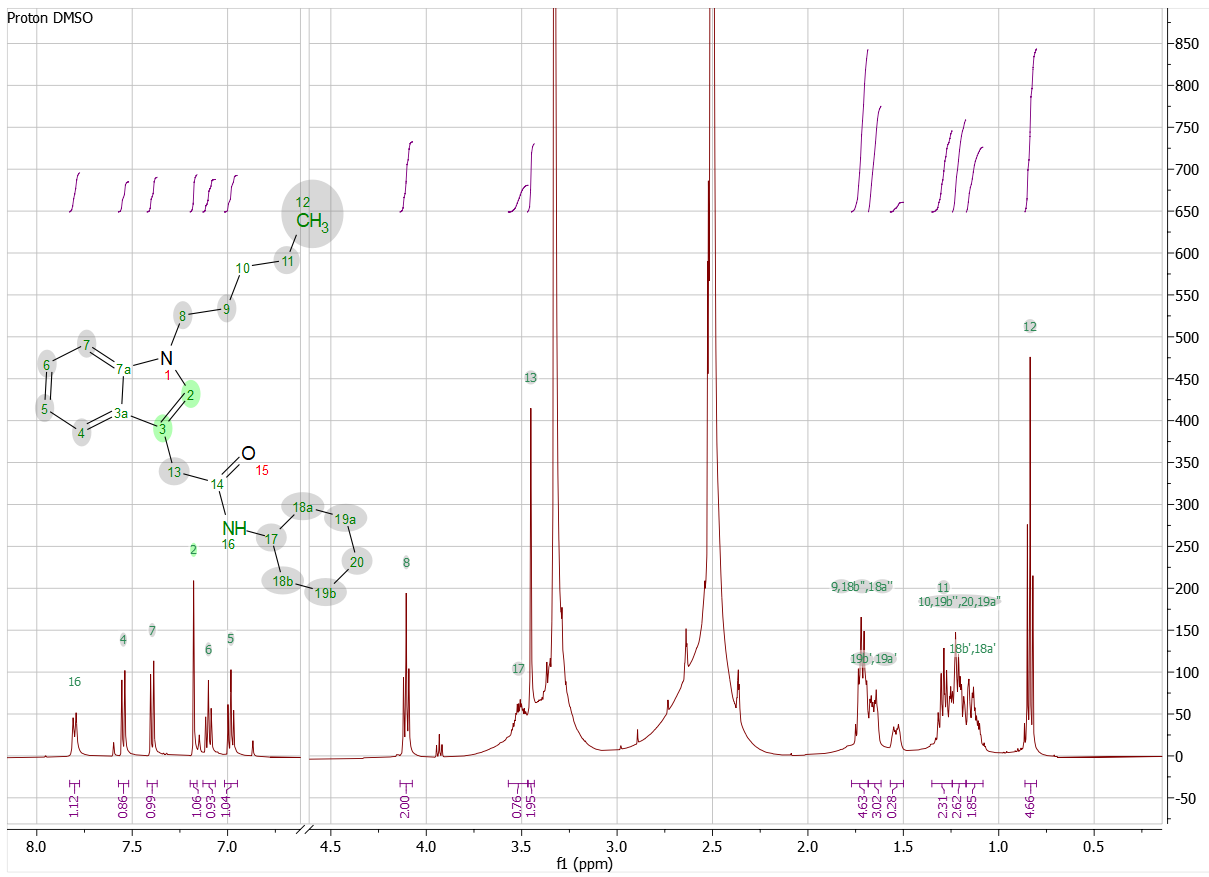


**Figure S6.** ^1^H spectrum of CH-PIACA in DMSO-*d*_6_.


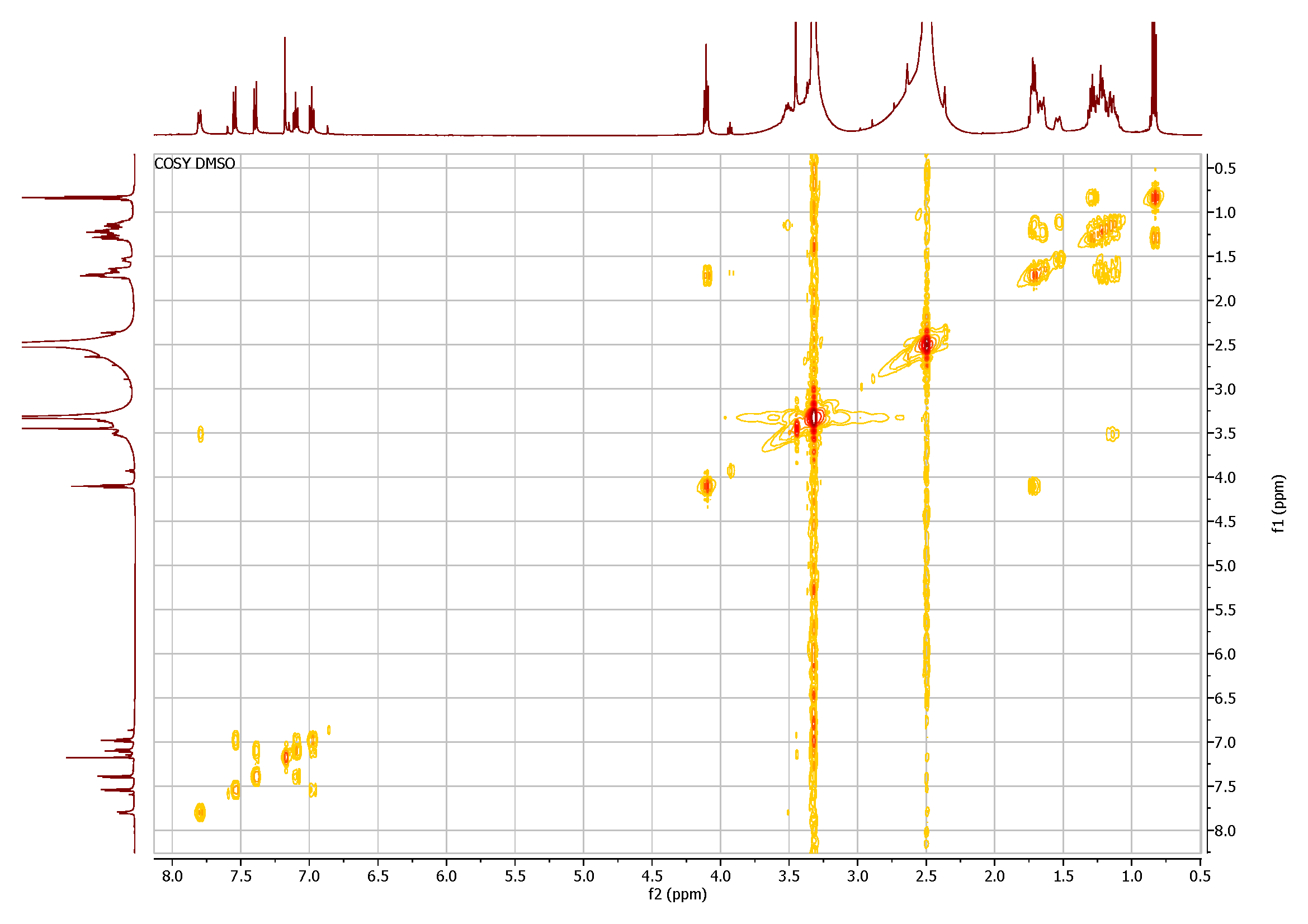


**Figure S7.** COSY spectrum of CH-PIACA in DMSO-*d*_6_.


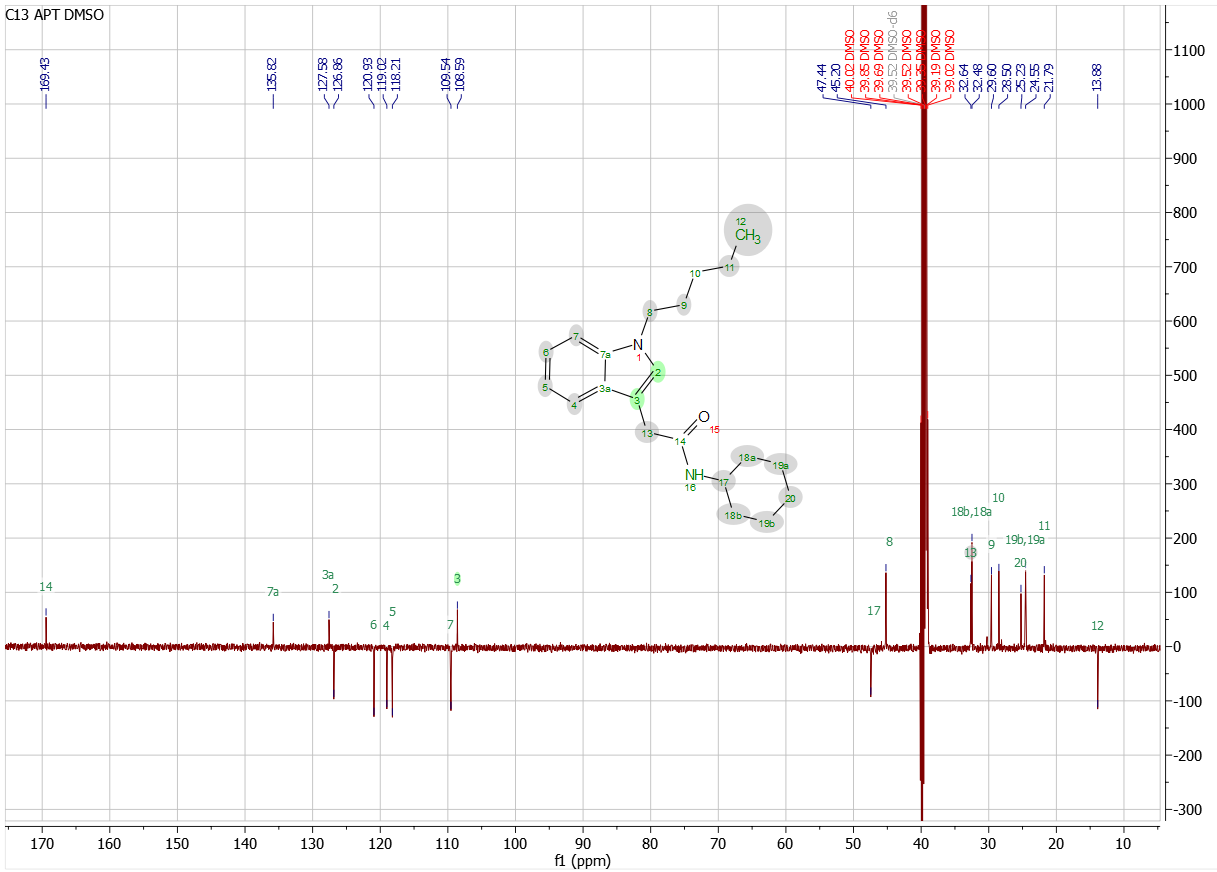


**Figure S8.** APT^13^C spectrum of CH-PIACA in DMSO-*d*_6_.


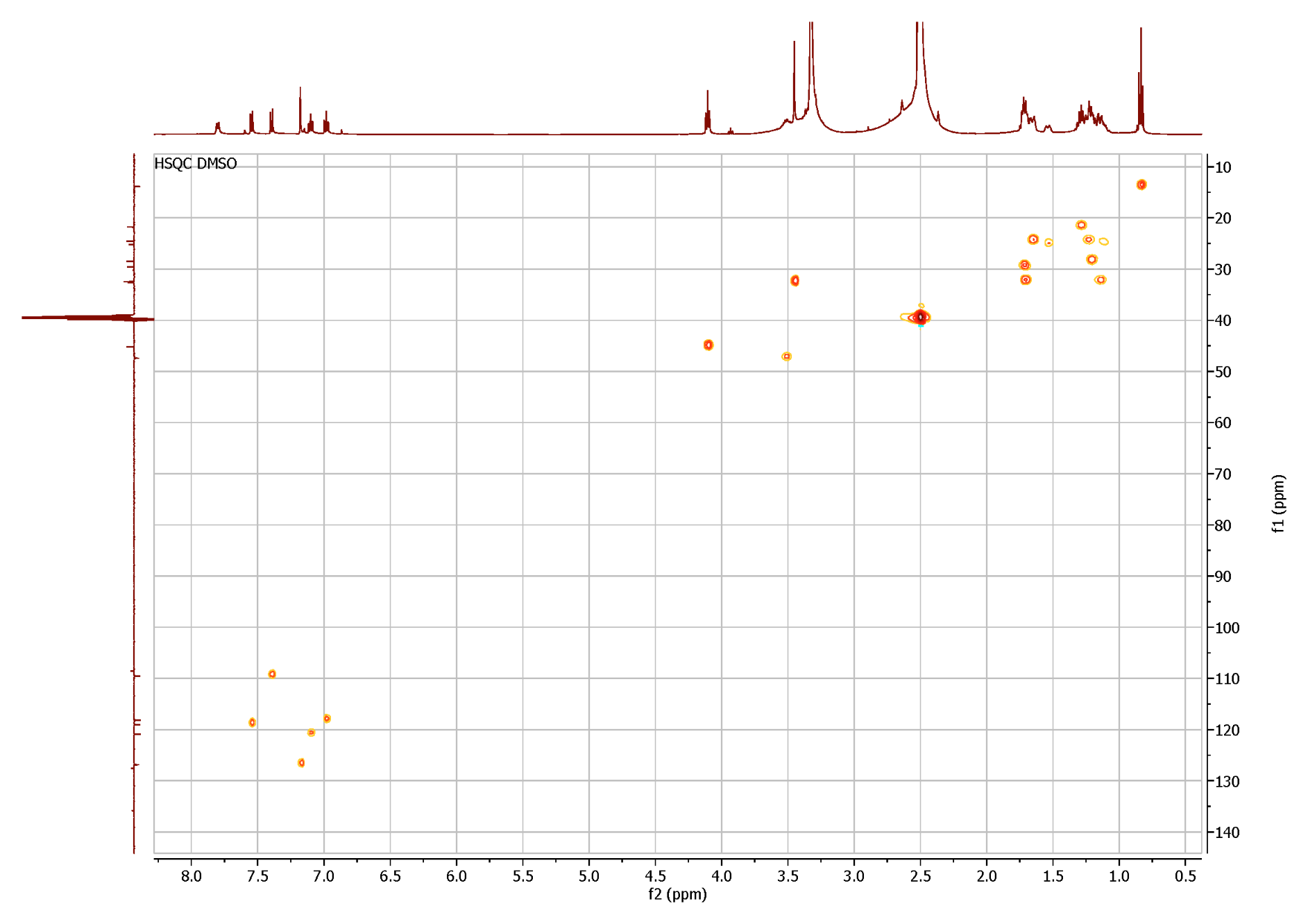


**Figure S9.** HSQC spectrum of CH-PIACA in DMSO-*d*_6_.


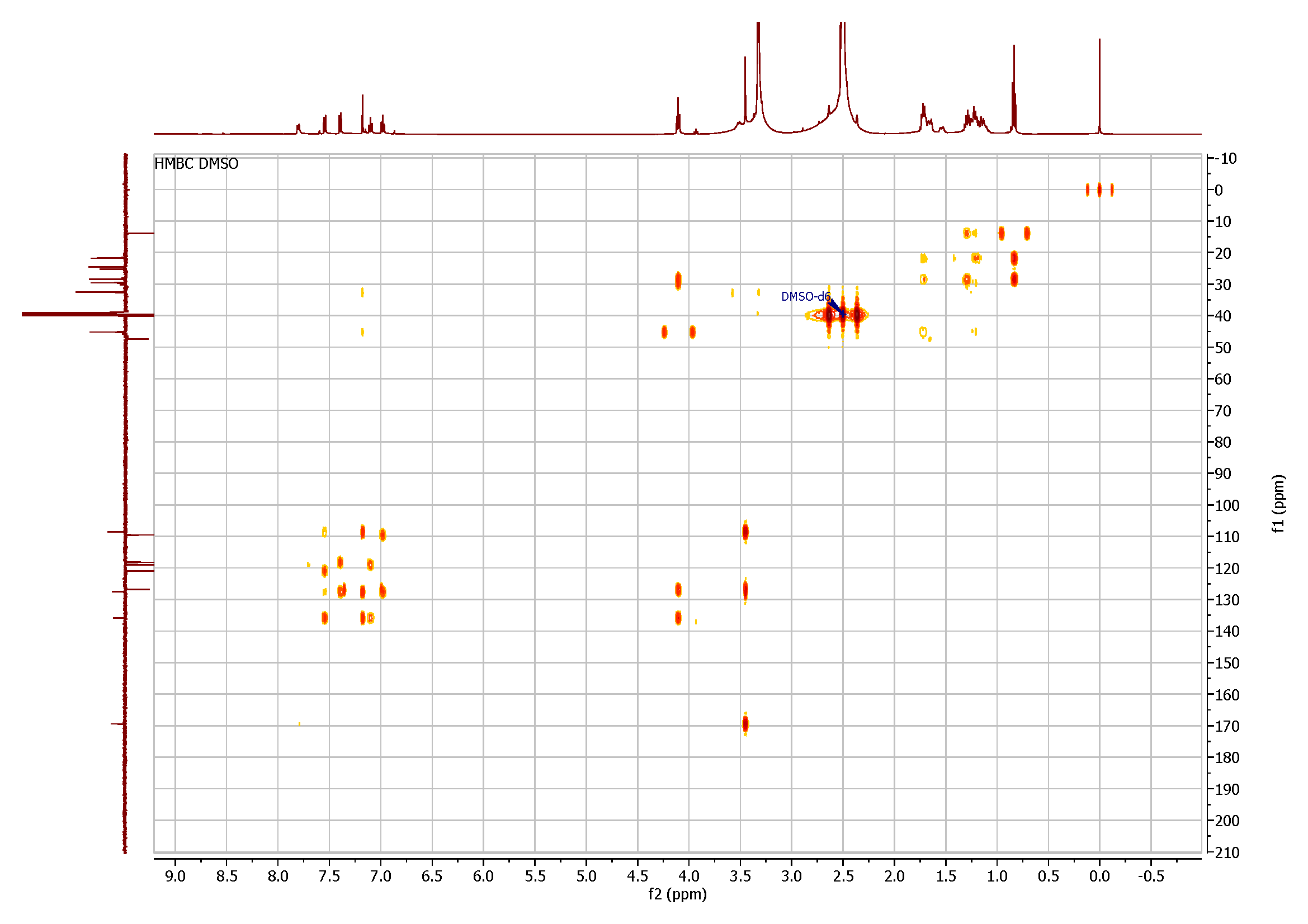


**Figure S10.** HMBC spectrum of CH-PIACA in DMSO-*d*_6_.


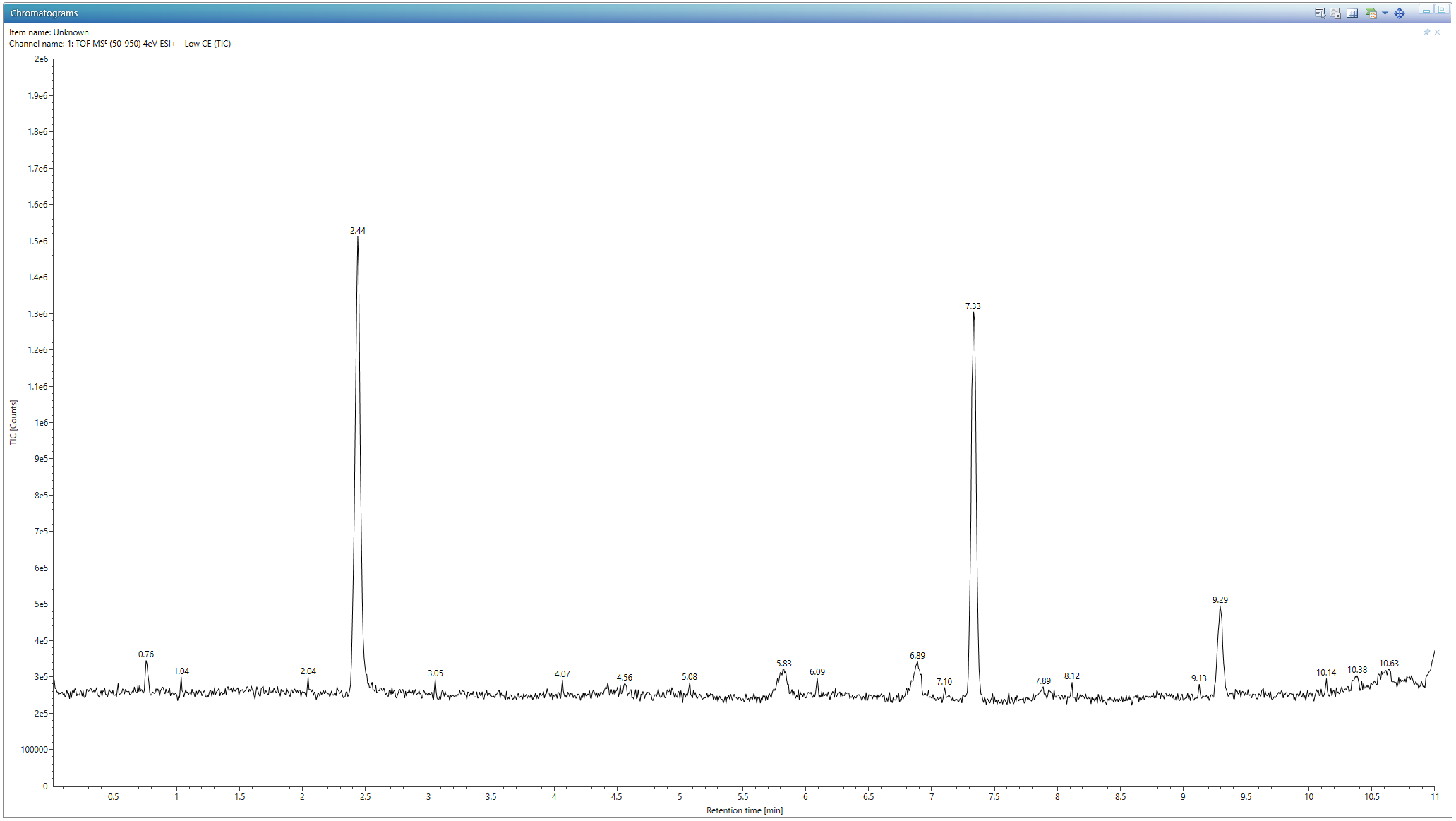


Unknown 1

Unknown 2

Unknown 3

Unknown 4

Internal

standard

**Figure S11. UPLC-QTOF-MS TIC of the seized material**


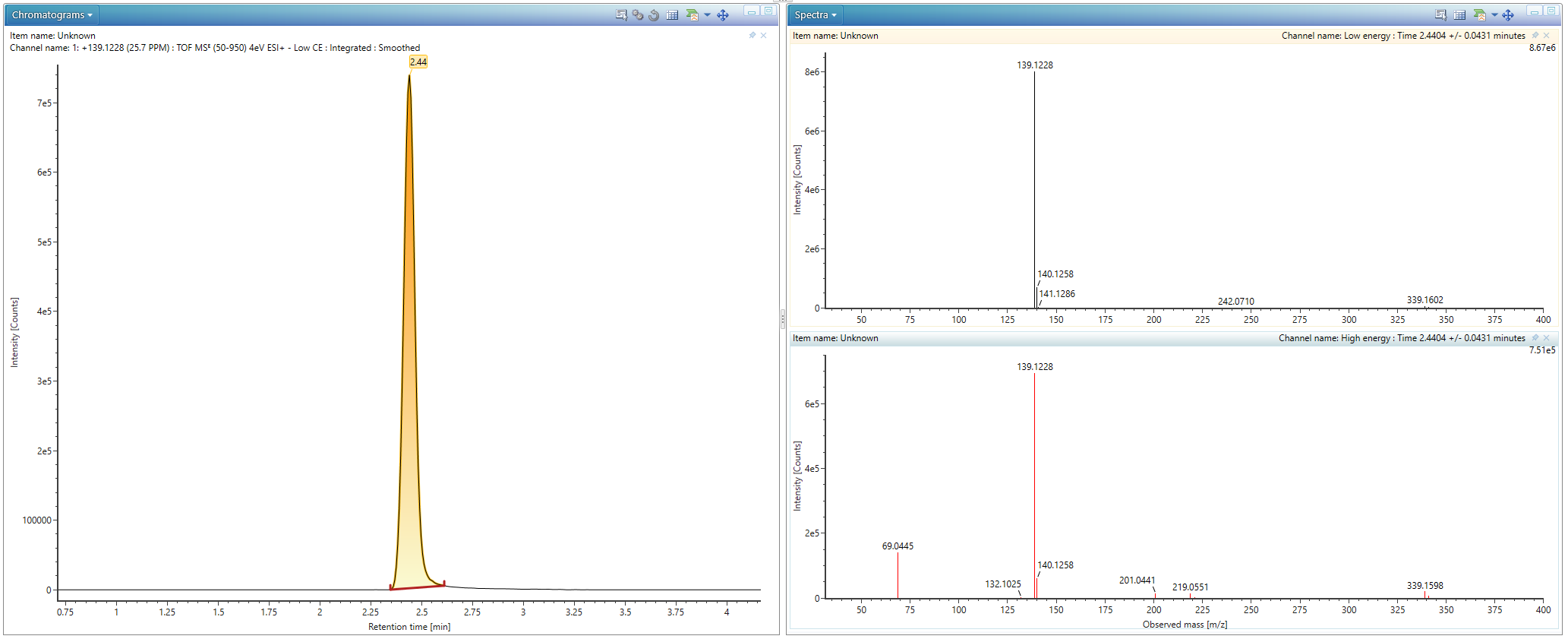


**Figure S12. ESI-MS of the unknown peak at 2.44 min**


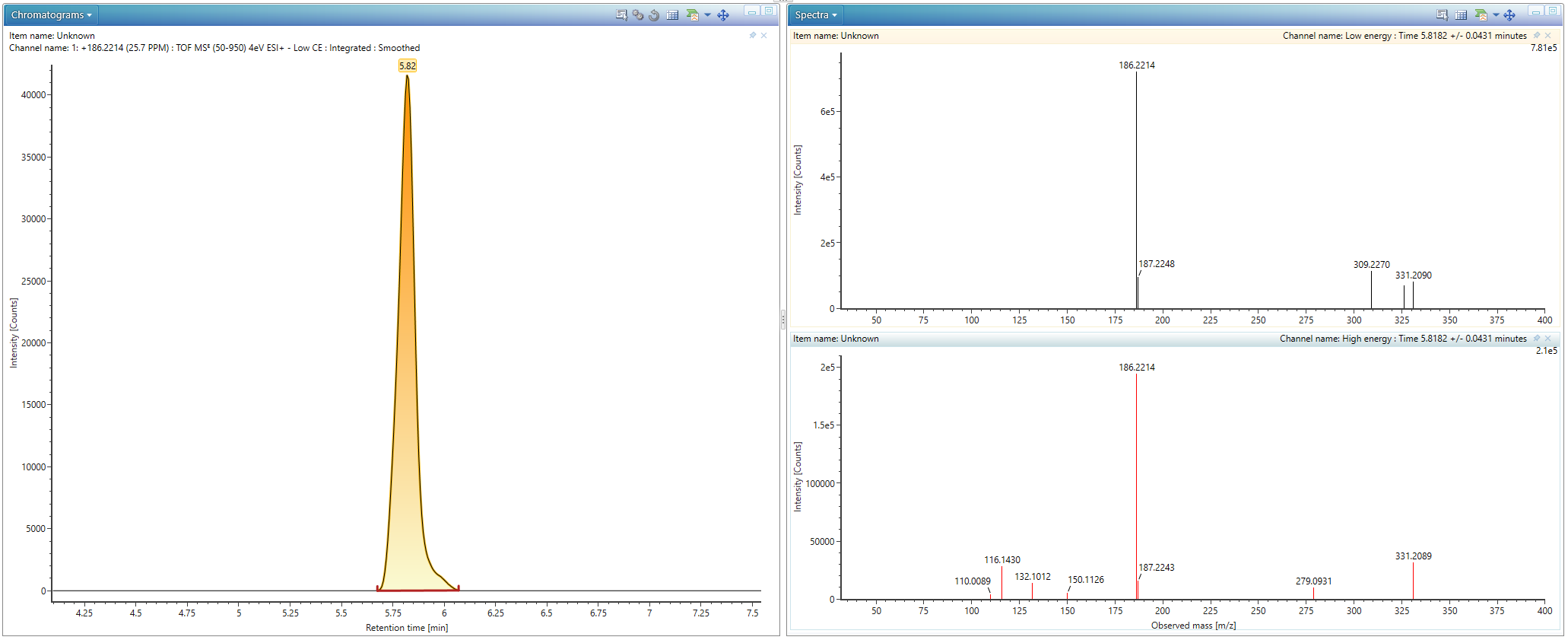


**Figure S13. ESI-MS of the unknown peak at 5.82 min**


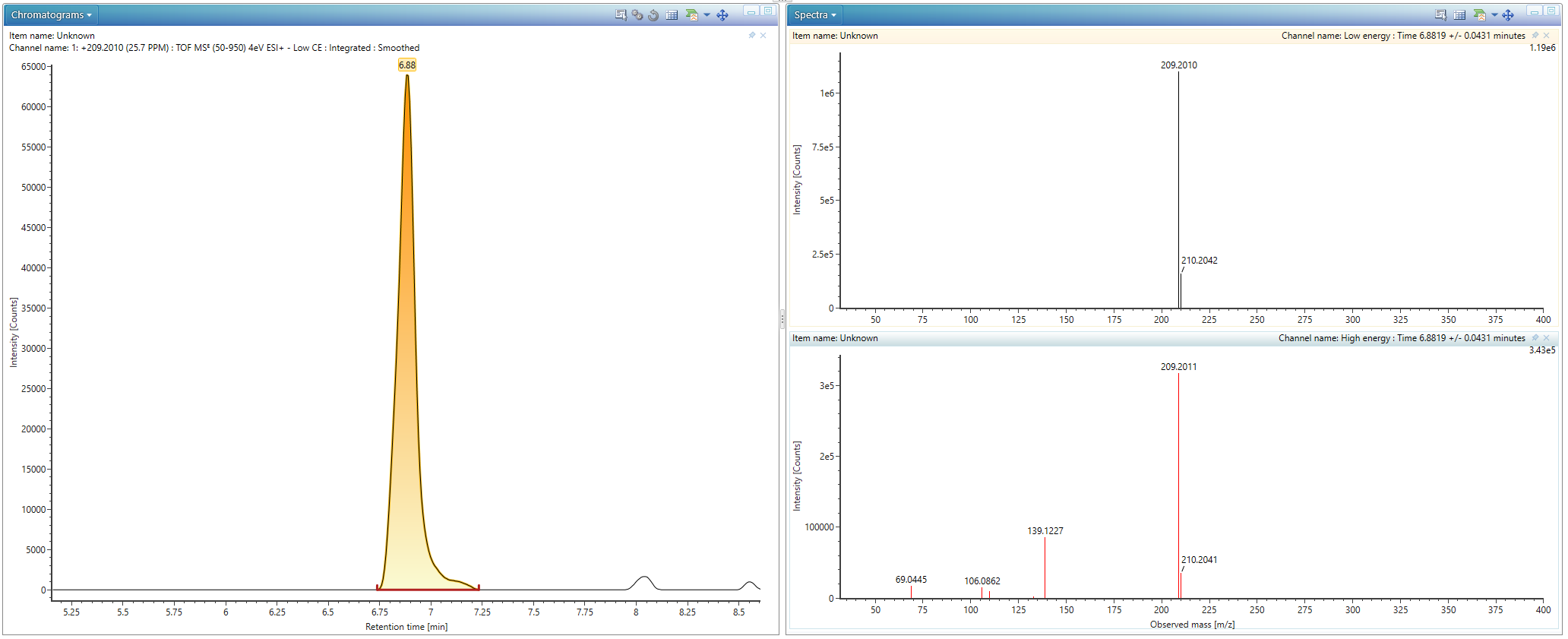


**Figure S14. ESI-MS of the unknown peak at 6.88 min**

**
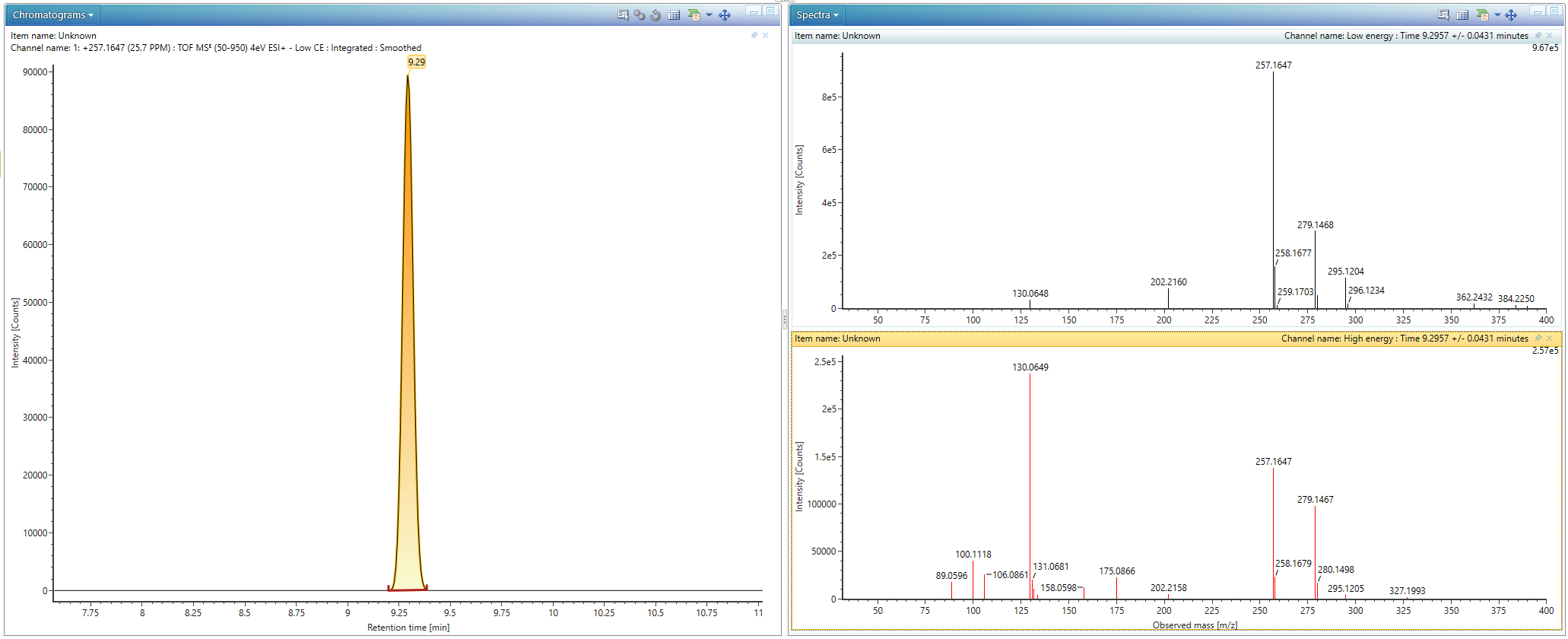
**

**Figure S15. ESI-MS of the unknown peak at 9.29 m**
